# Vibration and sound radiation of submerged finite cylindrical shells with pre-stress

**DOI:** 10.1038/s41598-019-47809-x

**Published:** 2019-08-05

**Authors:** Yongjin Guo, Hongdong Wang, Hong Yi

**Affiliations:** MOE Key Laboratory of Marine Intelligent Equipment and System, State Key Laboratory of Ocean Engineering, Shanghai Jiao Tong University, Shanghai, 200240 PR China

**Keywords:** Mechanical engineering, Aerospace engineering

## Abstract

Theoretical solutions of vibration and sound radiation of submerged finite cylindrical shells with pre-stress were derived by using a modal summation method. Motion equations of cylindrical shells with pre-stress were established on the basis of Flügge theory. Additional impedance caused by pre-stress was added to the basic vibration equation. Pre-stress was expressed by uniform and trigonometric forms to obtain the sound radiation power, mean quadratic velocity and radiation efficiency of cylindrical shells. Numerical calculation was conducted to study the effects of tensile and compressive stresses, stress direction, value size and distribution on vibration and sound radiation of cylindrical shells. This study may provide a reference for controlling sound radiation of underwater vehicles.

## Introduction

Vibration and sound radiation characteristics have important influences on the concealment and competitiveness of underwater vehicles whose basic structural form is a cylindrical shell. The structural noise of vehicles is from the sound radiation of surrounding fluid medium caused by shell vibration. Stepanishen^1^ studied the vibration and sound radiation of a cylindrical shell in fluid by modal superposition and radiation impedance method. Sandman^2,3^ defined the modal vibration velocity coefficients of a simply supported finite cylindrical shell with fluid loads. An effective numerical calculation method was proposed to calculate the integral of radiation impedance. Laulagnet and Guyader^4,5^ systematically studied the sound radiation performance of a finite ring-stiffened cylindrical shell. Jafari and Bagheri^6^ investigated the free vibration of cylindrical shells with circumferential stiffeners using analytical, experimental and finite element methods. Yan *et al*.^7^ studied the characteristics of vibrational power flow propagation in an infinite periodic ring-stiffened cylindrical shell immersed in water using space harmonic analysis. Liu *et al*.^8^ investigated the input power flow for an infinite ring-stiffened cylindrical shell submerged in fluid that was induced by a cosine harmonic circumferential line force under a uniform external hydrostatic pressure field condition. Zhou^9^ analysed the vibration and stability of ring-stiffened shells that convey fluid. The effects of fluid velocity, Young’s modulus, size and number of the ring stiffeners on the natural frequency and instability characteristics were also examined.

The aforementioned studies are all based on the ideal cylindrical shell. However, pre-stress may be generated during the production and work processes due to welding, assembling and water pressure, which will change the structural dynamic characteristics. Many studies have been conducted to solve this problem. Armenàkas^10^ studied the effects of initial uniform circumferential and axial stress on the dynamic response of simply supported cylindrical shells. PENZES and Kraus^11^ derived the exact free vibration solution of cylindrical shells with initial torque, pressure, axial force and rotational effect. Sivadas and Ganesan^12^ studied the free vibration of cylindrical shells with variable thickness and uniform circumferential external pressure. Liu and Chen^13^ studied the effect of weld residual stress on the free vibration characteristics of cylindrical shells. Yang *et al*.^14^ investigated the effect of welding residual stress on the vibration of underwater cylindrical shells by considering fluid structure interaction.

Generally, existing research mainly focuses on calculating the natural frequency and dynamic response of pre-stressed structures. In this study, the theoretical solutions of vibration and sound radiation of submerged finite cylindrical shells with pre-stress were derived on the basis of Flügge theory. Pre-stress was expressed by uniform and trigonometric forms to obtain the sound radiation power, mean quadratic velocity and radiation efficiency of cylindrical shells. Afterwards, the effects of tensile and compressive stresses, stress direction, value size and distribution on vibration and sound radiation of cylindrical shells are presented.

## Mathematical Modelling

**Figure 1:**
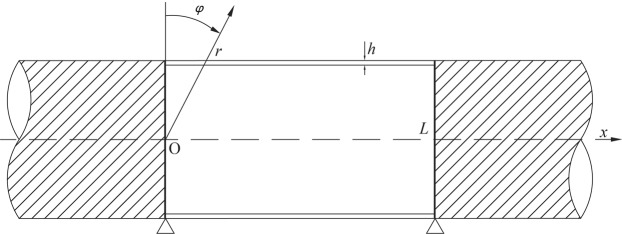
Cylindrical shell model.

Here, the cylindrical shell (Fig. 1), whose thickness divided by radius is less than 5%, is thin with uniform thickness *h*, radius *a*, length *L*, mass density ${\rho }_{p}$, modulus of elasticity *E* and Poisson’s ratio $\nu$. This shell is submerged in infinite water domain in which the sound velocity is ${c}_{0}$, and the fluid density is ${\rho }_{0}$. The axial, circumferential and radial displacements of the shell are represented by *u*, *v* and *w*, respectively.

Only the radial displacement *w* contributes to the sound field when the acoustic medium is surrounding the shell. The coupled vibration of the cylindrical shell in water is solved by the modal expansion method while ignoring the time factor ${e}^{-i\omega t}$. The rigid cylindrical baffles with semi-infinite length are connected at both ends of the shell. The symmetric excitation can be expressed as follows:

1 $$\{\begin{array}{c}u(\phi ,x)=\sum _{m=1}^{M}\,\sum _{n=0}^{N}{U}_{mn}\,\cos (n\phi )\,\cos ({k}_{m}x)\\ v(\phi ,x)=\sum _{m=1}^{M}\,\sum _{n=0}^{N}{V}_{mn}\,\sin (n\phi )\,\sin ({k}_{m}x)\\ w(\phi ,x)=\sum _{m=1}^{M}\,\sum _{n=0}^{N}{W}_{mn}\,\cos (n\phi )\,\sin ({k}_{m}x)\end{array},$$

where ${k}_{m}$ is determined by boundary conditions, and *m* and *n* are the axial and circumferential mode numbers, respectively. Assuming that the shell is simply supported, ${k}_{m}=m\lambda$ and $\lambda =\pi /L$ are obtained. The radial excitation force and surface sound pressure of the shell can be expanded as follows:

2 $$\{\begin{array}{c}f(\phi ,x)\\ p(a,\phi ,x)\end{array}\}=\sum _{m=1}^{M}\,\sum _{n=0}^{N}\{\begin{array}{c}{f}_{mn}\\ {p}_{mn}\end{array}\}\cos \,n\phi \,\cos \,{k}_{m}x.$$

The expansion coefficients are

3 $$\{\begin{array}{c}{f}_{mn}\\ {p}_{mn}\end{array}\}=\frac{{\varepsilon }_{n}}{\pi L}{\int }_{0}^{2\pi }{\int }_{0}^{L}\{\begin{array}{c}f(\phi ,x)\\ p(a,\phi ,x)\end{array}\}\cos \,n\phi \,\cos \,{k}_{m}x{\rm{d}}\phi {\rm{d}}x.$$

The modal equation is obtained by substituting Eqs (1) and (2) into the vibration equation of the shell, as shown as follows:

4 $${{\boldsymbol{A}}}_{mn}{{\boldsymbol{X}}}_{{mn}}=\frac{{a}^{2}}{{\rho }_{p}{c}_{p}^{2}h}(\begin{array}{c}0\\ 0\\ {f}_{mn}-{p}_{mn}\end{array}),$$

where ${{\boldsymbol{X}}}_{{mn}}{\boldsymbol{=}}{\{{{\boldsymbol{U}}}_{{mn}}{\boldsymbol{,}}{{\boldsymbol{V}}}_{{mn}}{\boldsymbol{,}}{{\boldsymbol{W}}}_{{mn}}\}}^{T}$. The classic thin shell theory includes Donnel equation, Flügge equation, etc. The Flügge equation has a higher precision. According to the Flügge shell equation^15^, the coefficient matrix is

5 $${{\boldsymbol{A}}}_{mn}=(\begin{array}{ccc}{a}_{11} & {a}_{12} & {a}_{13}\\ {a}_{21} & {a}_{22} & {a}_{23}\\ {a}_{31} & {a}_{32} & {a}_{33}\end{array}).$$

The elements of the matrix are

6 $$\{\begin{array}{c}{a}_{11}={{k}_{m}}^{2}{a}^{2}+\frac{1}{2}{n}^{2}(1+{\beta }^{2})(1-\nu )-{{\rm{\Omega }}}^{2}\\ {a}_{12}={a}_{21}=\frac{1}{2}{k}_{m}an(1+\nu )\\ {a}_{13}={a}_{31}={k}_{m}a\nu +{\beta }^{3}{{k}_{m}}^{3}{a}^{3}-\frac{1}{2}{n}^{2}{\beta }^{2}{k}_{m}a(1-\nu )\\ {a}_{22}=\frac{1}{2}{{k}_{m}}^{2}{a}^{2}(1-\nu )+{n}^{2}+\frac{3}{2}{\beta }^{2}{{k}_{m}}^{2}{a}^{2}(1-\nu )-{{\rm{\Omega }}}^{2}\\ {a}_{23}={a}_{32}=n+\frac{1}{2}{\beta }^{2}n{{k}_{m}}^{2}{a}^{2}(3-\nu )\\ {a}_{33}=1+{\beta }^{2}-2{\beta }^{2}{n}^{2}+{\beta }^{2}{({{k}_{m}}^{2}{a}^{2}+{n}^{2})}^{2}-{{\rm{\Omega }}}^{2}\end{array},$$

where ${c}_{p}=\sqrt{E/(1-{\nu }^{2}){\rho }_{p}}$ is the longitudinal wave velocity of the plate expanded by the shell, ${\rm{\Omega }}=\omega a/{c}_{p}$, ${\beta }^{2}={h}^{2}/12{a}^{2}$ and $\omega$ is the circular frequency. On the basis of Eq. (4), the modal vibration velocity satisfies the following condition:

7 $${\dot{W}}_{mn}{Z}_{mn}^{M}={f}_{mn}-{p}_{mn},$$

where ${Z}_{mn}^{M}$ is the mechanical impedance of the cylindrical shell, as shown as follows:

8 $${Z}_{mn}^{M}=i{\rho }_{p}{c}_{p}\frac{h}{a}\frac{|{A}_{mn}|}{{\rm{\Omega }}({a}_{11}{a}_{22}-{a}_{12}{a}_{21})}.$$

The surface sound pressure caused by sound radiation load is:

9 $${p}_{mn}=\sum _{q}^{\infty }{\dot{W}}_{qn}{Z}_{qmn}^{a},$$

where ${Z}_{qmn}^{a}$ is the mutual radiation impedance between $(q,n)$ and $(m,n)$ order modes, as shown as follows:

10 $${Z}_{qmn}^{a}=\frac{4}{\pi L}{\int }_{-\infty }^{\infty }\frac{{k}_{q}{k}_{m}{(-1)}^{q+m}{\cos }^{2}(KL/2)}{({k}_{q}^{2}-{K}^{2})({k}_{m}^{2}-{K}^{2})}{Z}_{n}^{a}(K){\bf{d}}K,$$

where ${Z}_{n}^{a}(K)$ is the sound radiation impedance of *K* space, as shown as follows:

11 $${Z}_{n}^{a}(K)=\frac{i{\rho }_{0}\omega {{\rm{H}}}_{n}^{(1)}(\sqrt{{k}_{0}^{2}-{K}^{2}}a)}{\sqrt{{k}_{0}^{2}-{K}^{2}}{{\rm{H}}}_{n}^{(1)^{\prime} }(\sqrt{{k}_{0}^{2}-{K}^{2}}a)}.$$

The coupled vibration equation with sound radiation load is obtained as

12 $${\dot{W}}_{mn}{Z}_{mn}^{M}+\sum _{q}^{\infty }{\dot{W}}_{qn}{Z}_{qmn}^{a}={f}_{mn}.$$

The sound radiation power of the shell is

13 $$P(\omega )=\frac{1}{2}{\int }_{S}{\rm{Re}}\{p(a,\phi ,x){\dot{w}}^{\ast }(\phi ,x)\}{\rm{d}}S=\frac{S}{4}\mathrm{Re}\{\sum _{q=1}^{Q}\,\sum _{m=1}^{M}\,\sum _{n=0}^{N}\frac{1}{{\varepsilon }_{n}}{\dot{W}}_{qn}{Z}_{qmn}^{a}{\dot{W}}_{mn}^{\ast }\},$$

where *S* is the surface area of the shell. When $n=0$ and $n\ge 1$, ${\varepsilon }_{n}=1$ and ${\varepsilon }_{n}=2$, respectively. The mean quadratic velocity is

14 $$\langle \dot{w}(\phi ,x){\dot{w}}^{\ast }(\phi ,x)\rangle =\frac{1}{2S}{\int }_{S}\{\dot{w}(\phi ,x){\dot{w}}^{\ast }(\phi ,x)\}{\rm{d}}S=\frac{1}{4}\sum _{m=1}^{M}\,\sum _{n=0}^{N}\frac{1}{{\varepsilon }_{n}}{\dot{W}}_{mn}{\dot{W}}_{mn}^{\ast }.$$

The radiation efficiency is

15 $$\sigma (\omega )=\frac{P(\omega )}{{\rho }_{0}{c}_{0}S\langle \dot{w}(\phi ,x){\dot{w}}^{\ast }(\phi ,x)\rangle }=\frac{\mathrm{Re}\{\sum _{q=1}^{Q}\,\sum _{m=1}^{M}\,\sum _{n=0}^{N}\frac{1}{{\varepsilon }_{n}}{\dot{W}}_{qn}{Z}_{qmn}^{a}{\dot{W}}_{mn}^{\ast }\}}{{\rho }_{0}{c}_{0}\sum _{m=1}^{M}\,\sum _{n=0}^{N}\frac{1}{{\varepsilon }_{n}}{\dot{W}}_{mn}{\dot{W}}_{mn}^{\ast }}.$$

## Effect of Pre-Stress

Liu and Chen^13^ indicated that the vibration equation of cylindrical shell with pre-stress can be expressed as

16 $$\{\begin{array}{c}{L}_{1}(u,v,w)+{C}_{1}(u,v,w,{\sigma }_{x}^{r},{\sigma }_{\phi }^{r},{\tau }_{x\phi }^{r})=\frac{\rho h}{D}\frac{{\partial }^{2}u}{\partial {t}^{2}}\\ {L}_{2}(u,v,w)+{C}_{2}(u,v,w,{\sigma }_{x}^{r},{\sigma }_{\phi }^{r},{\tau }_{x\phi }^{r})=\frac{\rho h}{D}\frac{{\partial }^{2}v}{\partial {t}^{2}}\\ {L}_{3}(u,v,w)-{C}_{3}(u,v,w,{\sigma }_{x}^{r},{\sigma }_{\phi }^{r},{\tau }_{x\phi }^{r})=-\frac{\rho h}{D}\frac{{\partial }^{2}w}{\partial {t}^{2}}\end{array},$$

where ${L}_{1}(u,v,w)$, ${L}_{2}(u,v,w)$ and ${L}_{3}(u,v,w)$ are the displacement terms of the Flügge equation; $D=\frac{E{h}^{3}}{12(1-{\nu }^{2})}$ is the bending stiffness; and *C*_1_, *C*_2_ and *C*_3_ represent the coupling terms of structural stress and vibration, whose specific expressions are as follows:

$$\begin{array}{rcl}{C}_{1}(u,v,w,{\sigma }_{x}^{r},{\sigma }_{\phi }^{r},{\tau }_{x\phi }^{r}) & = & \frac{h}{D}[(\frac{\partial {\sigma }_{x}^{r}}{\partial x}+\frac{\partial {\tau }_{x\phi }^{r}}{a\partial \phi })\frac{\partial u}{\partial x}+(\frac{\partial {\sigma }_{\phi }^{r}}{{a}^{2}\partial \phi }+\frac{\partial {\tau }_{x\phi }^{r}}{a\partial x})\frac{\partial u}{\partial \phi }+{\sigma }_{x}^{r}\frac{{\partial }^{2}u}{\partial {x}^{2}}\\  &  & +\,2\frac{{\tau }_{x\phi }^{r}}{a}\frac{{\partial }^{2}u}{\partial x\partial \phi }+\frac{{\sigma }_{\phi }^{r}}{{a}^{2}}\frac{{\partial }^{2}u}{\partial {\phi }^{2}}],\end{array}$$

$$\begin{array}{rcl}{C}_{2}(u,v,w,{\sigma }_{x}^{r},{\sigma }_{\phi }^{r},{\tau }_{x\phi }^{r}) & = & \frac{h}{D}[(\frac{\partial {\sigma }_{\phi }^{r}}{{a}^{2}\partial \phi }+\frac{\partial {\tau }_{x\phi }^{r}}{a\partial x})\frac{\partial v}{\partial \phi }+(\frac{\partial {\tau }_{x\phi }^{r}}{a\partial \phi }+\frac{\partial {\sigma }_{x}^{r}}{\partial x})\frac{\partial v}{\partial x}+{\sigma }_{\phi }^{r}\frac{{\partial }^{2}v}{{a}^{2}\partial {\phi }^{2}}\\  &  & +\,\frac{2{\tau }_{x\phi }^{r}}{a}\frac{{\partial }^{2}v}{\partial \phi \partial x}+{\sigma }_{x}^{r}\frac{{\partial }^{2}v}{\partial {x}^{2}}+\frac{\partial {\sigma }_{\phi }^{r}}{\partial \phi }\frac{w}{{a}^{2}}\\  &  & +\,\frac{\partial {\tau }_{x\phi }^{r}}{\partial x}\frac{w}{a}+\frac{{\sigma }_{\phi }^{r}}{{a}^{2}}\frac{\partial w}{\partial \phi }+\frac{{\tau }_{x\phi }^{r}}{a}\frac{\partial w}{a\partial x}],\end{array}$$

$$\begin{array}{rcl}{C}_{3}(u,v,w,{\sigma }_{x}^{r},{\sigma }_{\phi }^{r},{\tau }_{x\phi }^{r}) & = & \frac{h}{D}[-\frac{\partial {\sigma }_{\phi }^{r}}{\partial \phi }\frac{v}{{a}^{2}}-\frac{\partial {\tau }_{x\phi }^{r}}{\partial x}\frac{v}{a}-\frac{{\sigma }_{\phi }^{r}}{{a}^{2}}\frac{\partial v}{\partial \phi }-\frac{{\tau }_{x\phi }^{r}}{a}\frac{\partial v}{\partial x}\\  &  & +\,(\frac{\partial {\sigma }_{\phi }^{r}}{{a}^{2}\partial \phi }+\frac{\partial {\tau }_{x\phi }^{r}}{a\partial x})\frac{\partial w}{\partial \phi }+(\frac{\partial {\sigma }_{x}^{r}}{\partial x}+\frac{\partial {\tau }_{x\phi }^{r}}{a\partial \phi })\frac{\partial w}{\partial x}\\  &  & +\,{\sigma }_{x}^{r}\frac{{\partial }^{2}w}{\partial {x}^{2}}+2{\tau }_{x\phi }^{r}\frac{{\partial }^{2}w}{a\partial x\partial \phi }+{\sigma }_{\phi }^{r}\frac{{\partial }^{2}w}{{a}^{2}\partial {\phi }^{2}}],\end{array}$$

where ${\sigma }_{x}^{r}$, ${\sigma }_{\phi }^{r}$ and ${\tau }_{x\phi }^{r}$ are the axial positive, circumferential positive and shear stresses, respectively.

The coupling terms (i.e. *C*_1_, *C*_2_ and *C*_3_) of stress vibration are added to the equations compared with the differential formula of the cylindrical shell without stress. When the cylindrical shell has no pre-stress, the terms *C*_1_, *C*_2_ and *C*_3_ are zero, and Eq. (16) is simplified as the classical motion differential formula of cylindrical shell. Substituting Eq. (1) into Eq. (16) and simplifying Eq. (14) by the trigonometric function’s orthogonality yield the following:

17 $$\begin{array}{l}[-\frac{12}{{h}^{2}}{(m\lambda )}^{2}-\frac{6(1-\mu )}{{a}^{2}{h}^{2}}{n}^{2}-\frac{1-\mu }{2{a}^{4}}{n}^{2}]{U}_{mn}+\frac{6(1+\mu )}{a{h}^{2}}nm\lambda {V}_{mn}\\ \,+[\frac{12\mu }{a{h}^{2}}m\lambda +\frac{{(m\lambda )}^{3}}{a}-\frac{1-\mu }{2{a}^{3}}m\lambda {n}^{2}]{W}_{mn}+\frac{2}{\pi l}{\int }_{0}^{2\pi }\,{\int }_{0}^{l}{C}_{1}\,\cos (n\phi )\cos (m\lambda x)d\phi dx\\ \,+\frac{\rho h{\omega }^{2}}{D}{U}_{mn}=0,\end{array}$$

18 $$\begin{array}{c}\frac{6(1+\mu )}{a{h}^{2}}nm\lambda {U}_{mn}-[\frac{12{n}^{2}}{{a}^{2}{h}^{2}}+\frac{6(1-\mu )}{{h}^{2}}{(m\lambda )}^{2}+\frac{3(1-\mu )}{2{a}^{2}}{(m\lambda )}^{2}]{V}_{mn}\\ \,-\,[\frac{12n}{{a}^{2}{h}^{2}}+\frac{3-\mu }{2{a}^{2}}{(m\lambda )}^{2}n]{W}_{mn}+\frac{2}{\pi l}{\int }_{0}^{2\pi }{\int }_{0}^{l}{C}_{2}\,\sin (n\phi )\sin (m\lambda x)d\phi dx+\frac{\rho h{\omega }^{2}}{D}{V}_{mn}=0,\end{array}$$

19 $$\begin{array}{c}[\frac{12\mu }{a{h}^{2}}m\lambda +\frac{{(m\lambda )}^{3}}{a}-\frac{1-\mu }{2{a}^{3}}m\lambda {n}^{2}]{U}_{mn}-[\frac{12n}{{a}^{2}{h}^{2}}+\frac{3-\mu }{2{a}^{2}}{(m\lambda )}^{2}n]\\ \,{V}_{mn}-[\frac{12}{{a}^{2}{h}^{2}}+{({m}^{2}{\lambda }^{2}+\frac{{n}^{2}}{{a}^{2}})}^{2}+\frac{1}{{a}^{4}}-2\frac{{n}^{2}}{{a}^{4}}]{W}_{mn}\\ \,+\,\frac{2}{\pi l}{\int }_{0}^{2\pi }{\int }_{0}^{l}{C}_{3}\,\cos (n\phi )\sin (m\lambda x)d\phi dx+\frac{\rho h{\omega }^{2}}{D}{W}_{mn}=0.\end{array}$$

For expression simplification, ${K}_{1}={\int }_{0}^{2\pi }{\int }_{0}^{l}{C}_{1}\,\cos (n\phi )\cos (m\lambda x)dxd\phi$, ${K}_{2}={\int }_{0}^{2\pi }{\int }_{0}^{l}{C}_{2}\,\sin (n\phi )\sin (m\lambda x)dxd\phi$ and ${K}_{3}={\int }_{0}^{2\pi }{\int }_{0}^{l}{C}_{3}\,\sin (n\phi )\sin (m\lambda x)dxd\phi$. Different pre-stress forms lead to varying values. The results of two typical pre-stress distribution forms are presented as follows.

(1) Assume that the shear stress of the cylindrical shell is ${\tau }_{x\phi }^{r}=0$. Pre-stresses ${\sigma }_{x}^{r}$ and ${\sigma }_{\phi }^{r}$ are uniformly distributed throughout the entire shell, which can be expressed as follows:

20 $$\{\begin{array}{c}{\sigma }_{x}^{r}={\sigma }_{0}^{rx}\\ {\sigma }_{\phi }^{r}={\sigma }_{0}^{r\phi }\end{array}.$$

Here, Eq. (21) can be obtained by the trigonometric function’s orthogonality and substituting the pre-stress into ${K}_{1}$, ${K}_{2}$ and ${K}_{3}$.

21 $${{\boldsymbol{A}}}_{mn}^{^{\prime} }{{\boldsymbol{X}}}_{{mn}}=0,$$

where ${{\boldsymbol{A}}}_{mn}^{\text{'}}={{\boldsymbol{A}}}_{mn}+{{\boldsymbol{R}}}_{mn}$ is the coefficient matrix of the shell considering the pre-stresses. In comparison with the shell without pre-stress, the pre-stress influence matrix is added, as shown as follows:

22 $${{\boldsymbol{R}}}_{mn}=\frac{{a}^{2}(1-{\nu }^{2})}{E}[\begin{array}{ccc}{R}_{11} & 0 & 0\\ 0 & {R}_{22} & {R}_{23}\\ 0 & {R}_{32} & {R}_{33}\end{array}],$$

where $${R}_{11}={k}_{m}^{2}{\sigma }_{0}^{rx}+\frac{{n}^{2}}{{a}^{2}}{\sigma }_{0}^{r\phi }$$, $${R}_{22}={k}_{m}^{2}{\sigma }_{0}^{rx}+\frac{{n}^{2}}{{a}^{2}}{\sigma }_{0}^{r\phi }$$, $${R}_{23}=-\,\frac{{n}^{2}}{{a}^{2}}{\sigma }_{0}^{r\phi }$$, $${R}_{32}={R}_{23}$$ and $${R}_{33}={R}_{22}$$.

(2) Assume that the shear stress of the cylindrical shell is ${\tau }_{x\phi }^{r}=0$. The pre-stress value changes in one direction only and can be expressed as follows:

23 $$\{\begin{array}{c}{\sigma }_{x}^{r}={\sigma }_{g}^{rx}\,\cos (g\lambda x)\\ {\sigma }_{\phi }^{r}={\sigma }_{g}^{r\phi }\,\cos (g\lambda x)\end{array},$$

where ${\sigma }_{g}^{rx}$ and ${\sigma }_{g}^{r\phi }$ are structural stress amplitudes, and *g* is a positive integer that represents the modal order corresponding to the stress.

The trigonometric function’s orthogonality yields Equation (24) by substituting the pre-stresses into ${K}_{1}$, ${K}_{2}$ and ${K}_{3}$.

24 $$({{\boldsymbol{\Lambda }}}_{n}+{{\bf{R}}}_{g}){{\bf{X}}}_{n}=0,$$

where ${{\bf{X}}}_{n}={\{{{\bf{X}}}_{1n}\cdots {{\bf{X}}}_{mn}\cdots {{\bf{X}}}_{Mn}\}}^{T}$, ${{\boldsymbol{\Lambda }}}_{n}$ is a diagonal matrix composed of the shell equation coefficients and ${{\bf{R}}}_{g}$ is the pre-stress influence matrix.

25 $${{\boldsymbol{\Lambda }}}_{n}=[\begin{array}{ccccc}{{\bf{A}}}_{1n} &  &  &  & \\  & \ddots  &  &  & \\  &  & {{\bf{A}}}_{mn} &  & \\  &  &  & \ddots  & \\  &  &  &  & {{\bf{A}}}_{Mn}\end{array}],$$

26 $${{\bf{R}}}_{g}=[\begin{array}{ccc}{{\bf{R}}}_{11}^{g} & \cdots  & {{\bf{R}}}_{1q}^{g}\\ \vdots  & \ddots  & \vdots \\ {{\bf{R}}}_{p1}^{g} & \cdots  & {{\bf{R}}}_{pq}^{g}\end{array}],$$

where non-zero partition matrix $${{\bf{R}}}_{pq}^{g}=\frac{{a}^{2}(1-{\nu }^{2})}{2E}[\begin{array}{ccc}{R}_{11}^{pq} & 0 & 0\\ 0 & {R}_{22}^{pq} & {R}_{23}^{pq}\\ 0 & {R}_{32}^{pq} & {R}_{33}^{pq}\end{array}]$$, and the non-zero elements of ${{\bf{R}}}_{pq}^{g}$ can be obtained as follows.

When $$p=m$$, $$q=m-g$$ and $$m-g > 0$$, $${R}_{11}^{pq}=(m-g)m{\lambda }^{2}{\sigma }_{g}^{rx}+\frac{{n}^{2}}{{a}^{2}}{\sigma }_{g}^{r\phi }$$, $${R}_{22}^{pq}={R}_{33}^{pq}=-\,{R}_{11}^{pq}$$, $${R}_{23}^{pq}=$$$$-\frac{n}{{a}^{2}}{\sigma }_{g}^{r\phi }$$ and $${R}_{32}^{pq}={R}_{23}^{pq}$$.

When $p=m$, $q=|m-g|$ and $m-g < 0$, ${R}_{11}^{pq}=(m-g)m{\lambda }^{2}{\sigma }_{g}^{rx}+\frac{{n}^{2}}{{a}^{2}}{\sigma }_{g}^{r\phi }$, ${R}_{22}^{pq}={R}_{33}^{pq}={R}_{11}^{pq}$, ${R}_{23}^{pq}=\frac{n}{{a}^{2}}{\sigma }_{g}^{r\phi }$ and ${R}_{32}^{pq}=\frac{n}{{a}^{2}}{\sigma }_{g}^{r\phi }$.

When $p=m$ and $q=m+g$, ${R}_{11}^{pq}=(m+g)m{\lambda }^{2}{\sigma }_{g}^{rx}+\frac{{n}^{2}}{{a}^{2}}{\sigma }_{g}^{r\phi }$, ${R}_{22}^{pq}={R}_{33}^{pq}={R}_{11}^{pq}$, ${R}_{23}^{pq}=\frac{n}{{a}^{2}}{\sigma }_{g}^{r\phi }$ and ${R}_{32}^{pq}={R}_{23}^{pq}$.

## Numerical Calculation and Result Discussion

**Figure 2:**
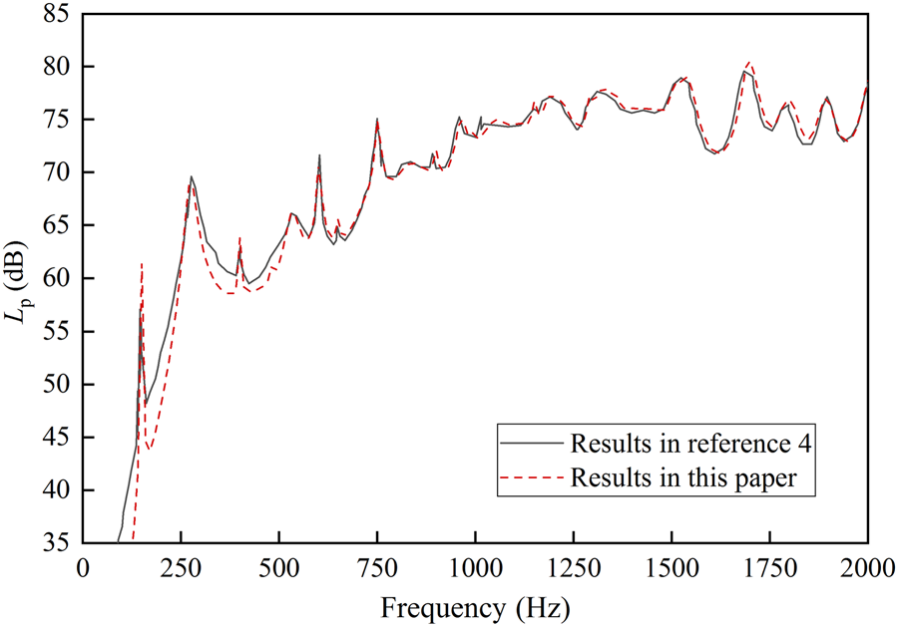
Sound radiation power comparison with ref.^4^.

The numerical calculation program is conducted on the basis of the theorical equations. In order to verify the accuracy of the program, the acoustic power results of the cylindrical shell without pre-stress are compared with the results in ref.^4^. A good agreement is shown in Fig. 2 between the results. The error in the low frequency band is caused by the difference of the calculated modal orders.

A simply supported cylindrical shell model is selected for the analysis. The basic parameters of the model are $a=0.4\,{\rm{m}}$, $L=1.2\,{\rm{m}}$ and $h=0.003\,{\rm{m}}$. The material parameters are ${\rho }_{p}={\rm{7800}}\,{\mathrm{kg}/m}^{{\rm{3}}}$, $E={\rm{2.1}}\times {{\rm{10}}}^{{\rm{11}}}\,{N/m}^{2}$ and $\nu =0.3$. The surrounding fluid is water, whose parameters are ${\rho }_{0}=1000\,{\mathrm{kg}/m}^{{\rm{3}}}$ and ${c}_{0}=1500\,m/s$. The excitation force concentrates at the locations of $\phi =0$ and $z=L/2$ with 1 N amplitude. Mutual coupling is ignored, and only $q=m$ items are considered. The calculated frequency band is 20~2000 Hz.

The sound radiation power level is defined as

27 $${L}_{p}(\omega )=10\,{\rm{lg}}\,\frac{P(\omega )}{{P}_{0}},$$

where ${P}_{0}={10}^{-12}{\rm{w}}$ is the reference sound power.

The mean quadratic velocity level is defined as

28 $${L}_{w}(\omega )=10\,{\rm{lg}}(\frac{\langle \dot{w}{\dot{w}}^{\ast }\rangle }{{\dot{w}}_{0}^{2}}),$$

where ${\dot{w}}_{0}=5\times {10}^{-8}\,m/s$ is the reference quadratic velocity.

The radiation efficiency level is

29 $${L}_{\sigma }(\omega )=10\,{\rm{lg}}(\sigma ).$$

### Influences of tensile and compressive pre-stresses

**Figure 3:**
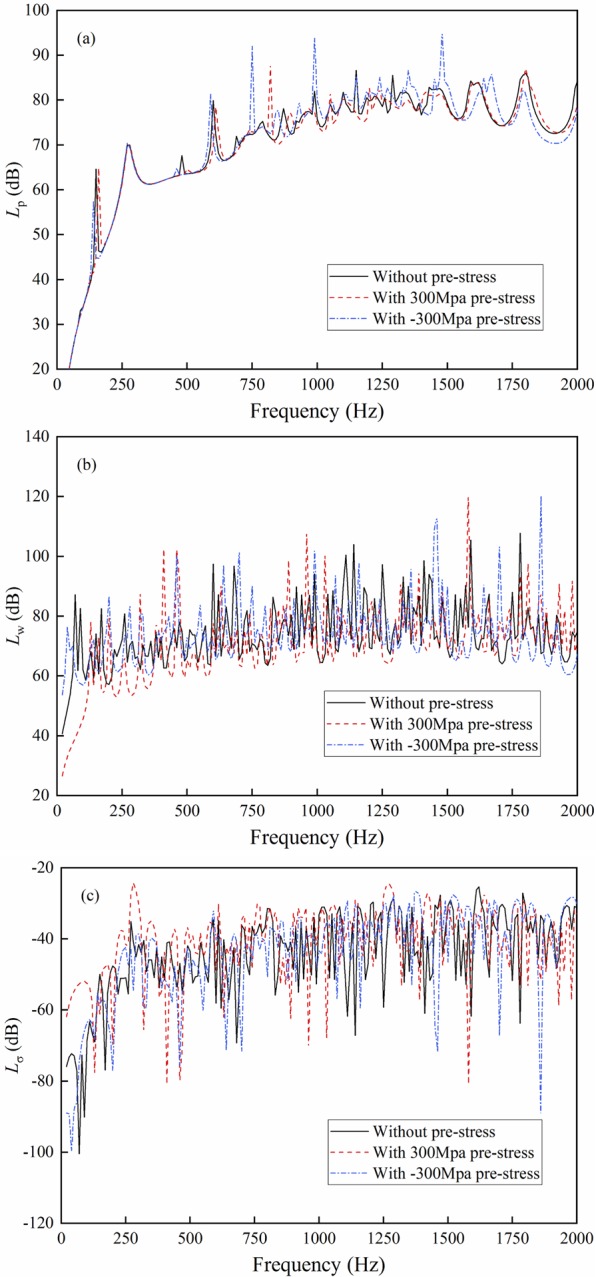
Result comparison with tensile and compressive pre-stresses. (**a**) Sound radiation power; (**b**) Mean quadratic velocity; (**c**) Radiation efficiency.

The uniform stresses of 300 MPa and −300 MPa, which often exist in the high-strength shipbuilding steel according to ref.^16^, are calculated as the basic stresses to apply to the cylindrical shell. The results are shown in Fig. 3.

As shown in Fig. 3(a), pre-stress does not considerably change the sound radiation power because it alters the mechanical impedance of the shell and does not affect radiation impedance. In the low frequency band, the sound radiation mainly comes from the low-order modes. The mechanical impedance and sound radiation changes caused by pre-stress are small. However, the existence of pre-stress changes the resonance characteristics of the shell, thereby resulting in some changes in the position and height of the peak. tensile and compressive stresses exhibit different effects on structural vibration and sound radiation. In some frequency bands, compressive stress leads to considerable peaks of structural sound radiation curve. The peak value of sound radiation with compressive stress moves to a lower frequency than that with tensile stress. This occurrence is due to compressive and axial stresses that decrease and increase the stiffness and natural frequency of the shell, respectively. As shown in Fig. 3(b), the tensile pre-stress reduces the mean quadratic velocity of the shell, particularly in the low frequency band (0~500 Hz). This occurrence is because of the increase of the total mechanical impedance of the shell and the decrease of the vibration speed with pre-stress. According to Eq. (15), an inevitable increase in radiation efficiency occurs because the sound radiation power does not change considerably, and the vibration speed evidently decreases, as shown in Fig. 3(c). The mean quadratic velocity of the shell with tensile stress is considerably smaller than that with compressive stress due to the stiffness increase, thereby leading to the increase of radiation efficiency.

### Influences of axial and circumferential pre-stresses

**Figure 4:**
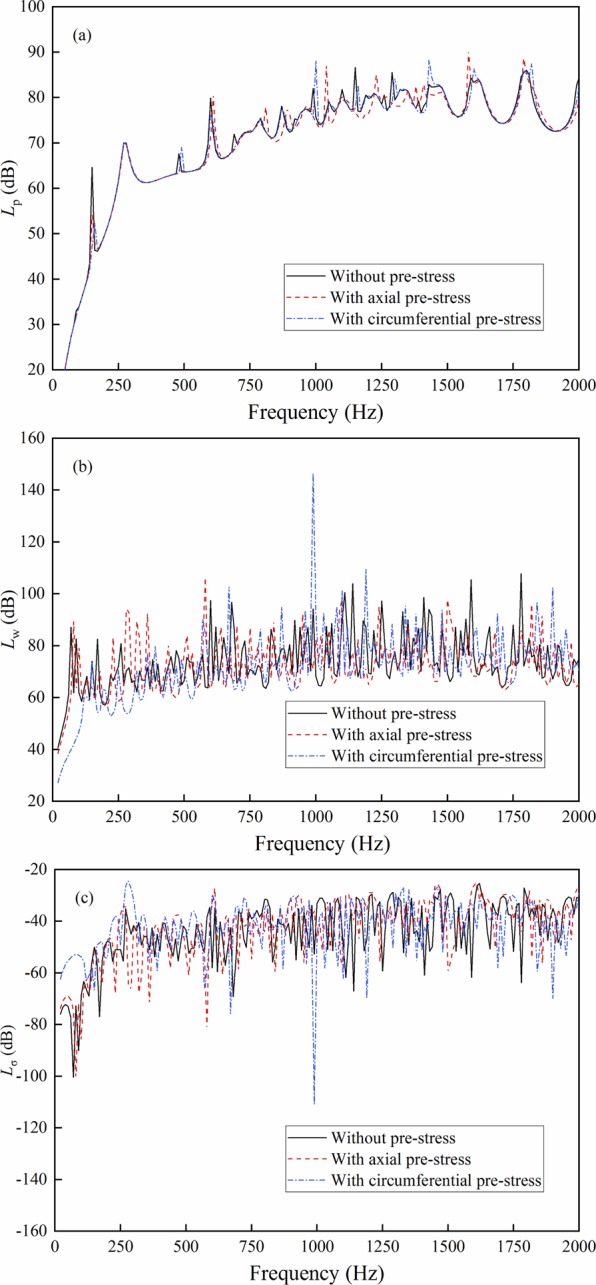
Result comparison with axial and circumferential pre-stress. (**a**) Sound radiation power; (**b**) Mean quadratic velocity; (**c**) Radiation efficiency.

The axial and circumferential uniform stresses are applied to the basic cylindrical shell. The stress value is 300 Mpa, and the results are shown in Fig. 4.

As depicted in Fig. 4(a), the circumferential and axial stresses demonstrate minimal effect on the sound acoustic power, which mainly change the extreme value at some frequencies. As shown in Fig. 4(b,c), relatively large changes are observed for the mean quadratic velocity and radiation efficiency. The circumferential stress exhibits a greater effect on the stiffness than the axial stress, which correspondingly changes the vibration characteristics of the shell. Simultaneously, the maximum mean quadratic velocity is generated for the mean quadratic velocity with circumferential stress due to the change of the mechanical impedance of the shell, which should be given attention during the structural design process.

### Influence of pre-stress value size

The axial and circumferential uniform stresses of 100, 300 and 500 Mpa are applied.

**Figure 5:**
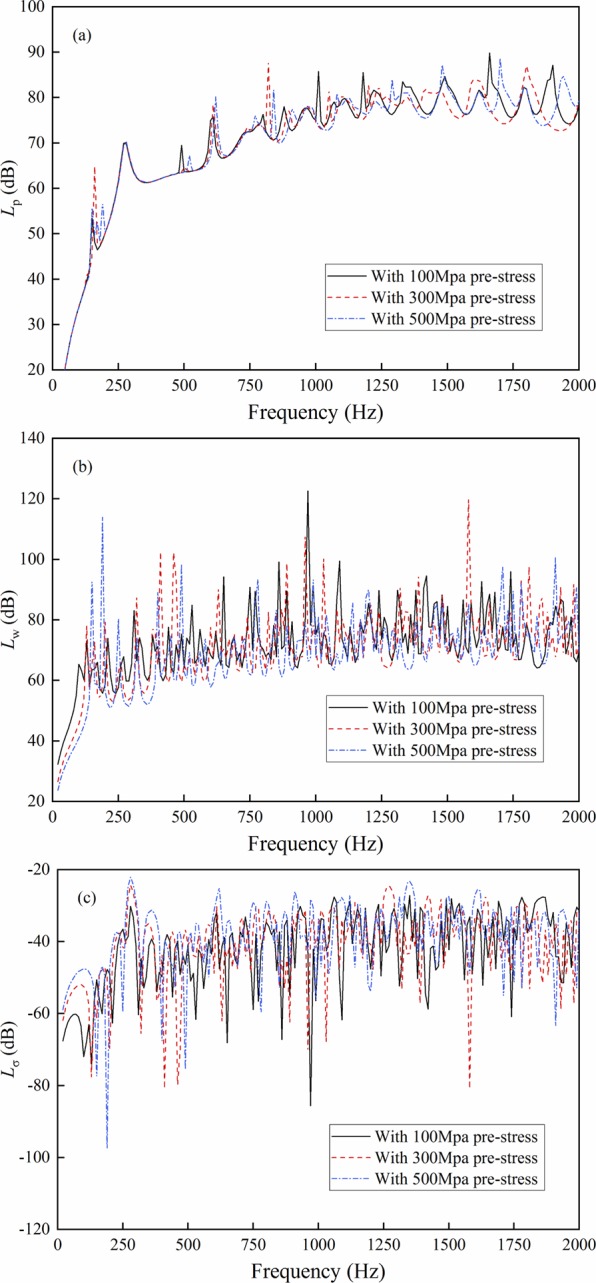
Result comparison with different pre-stress value size. (**a**) Sound radiation power; (**b**) Mean quadratic velocity; (**c**) Radiation efficiency.

As shown in Fig. 5, the pre-stress value size mainly changes the extreme value and makes the deviation of the sound radiation power curve. The influences of different pre-stress values on shell vibration and radiation efficiency vary in diverse frequency bands. This condition is due to the changes in the inherent characteristics of the cylindrical shell by pre-stress, including the natural frequencies and modes, thereby resulting in the changes of vibration and sound radiation properties. The change degree depends on the characteristics and pre-stress value of the shell.

### Influence of pre-stress distribution

**Figure 6:**
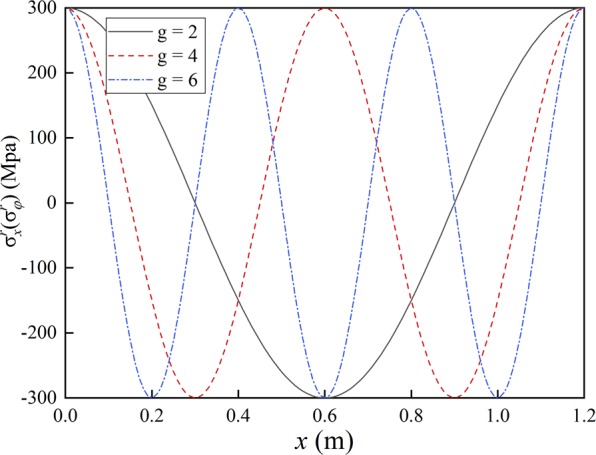
Pre-stress distribution.

**Figure 7:**
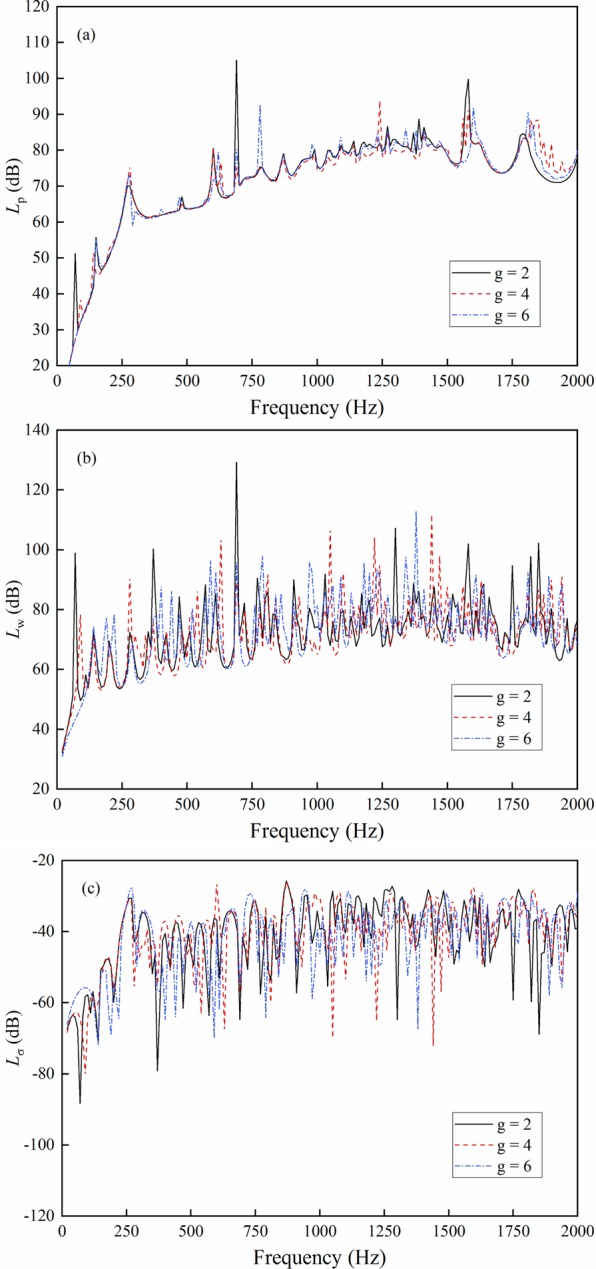
Result comparison with different pre-stress distributions. (**a**) Sound radiation power; (**b**) Mean quadratic velocity; (**c**) Radiation efficiency.

The trigonometric function is used to fit the pre-stress. The distribution forms of the pre-stress are shown in Fig. 6. The calculation results are shown in Fig. 7.

As shown in Fig. 7, different pre-stress distributions make diverse effects on the vibration and sound radiation of the cylindrical shell, thereby showing the effectiveness of the proposed method. The trigonometric function can effectively fit the pre-stress distribution and obtain accurate responses.

## Conclusions

The theoretical solutions of vibration and sound radiation of submerged finite cylindrical shells with pre-stress are obtained on the basis of Flügge theory and modal summation method. The effects of tensile and compressive stresses, stress direction, value size and distribution on vibration and sound radiation of the cylindrical shell are studied by the numerical calculation.

The results show that additional impedance caused by pre-stress is added to the basic vibration equation, which changes the structural vibration and sound radiation. Pre-stress changes the position and height of the peak of sound radiation power. Compressive stress decreases structural stiffness, whereas tensile stress increases it. The mean quadratic velocity with tensile stress is smaller than that with compressive stress. Circumferential stress has a greater effect on the stiffness than the axial stress. The pre-stress value size and distribution may affect the structural responses differently. The effect of pre-stress should be considered during designing, manufacturing and accurately predicting vibration and sound radiation of underwater vehicles.

## Data Availability

All data generated or analysed during this study are included in this published article.
